# From Disease to Pregnancy: Rethinking Cardiac Remodeling Through Fibroblast, Immune Cell, and Hormonal Interactions

**DOI:** 10.3390/cells15090778

**Published:** 2026-04-25

**Authors:** Emily B. Ruggiero, Wayne Carver, Daping Fan, Edie C. Goldsmith, Holly A. LaVoie

**Affiliations:** Department of Cell Biology and Anatomy, University of South Carolina School of Medicine, Columbia, SC 29208, USA; emily.ruggiero@uscmed.sc.edu (E.B.R.); wayne.carver@uscmed.sc.edu (W.C.); daping.fan@uscmed.sc.edu (D.F.)

**Keywords:** pregnancy, cardiac remodeling, fibrosis, extracellular matrix, immune system, fibroblasts, pregnancy hormones, prolactin

## Abstract

**Highlights:**

**What are the main findings?**

- The review emphasizes that pregnancy-associated cardiac remodeling is driven largely by hormonal signals—particularly estrogen, progesterone, prolactin, and relaxin—which promote an adaptive, reversible, and protective remodeling state, characterized by cardiomyocyte hypertrophy, enhanced angiogenesis, modulation of immune cell activation, and controlled extracellular matrix remodeling without net fibrosis.

**What are the implications of the main findings?**

- Focusing on non-myocyte cell populations and matrix dynamics, this review promotes pregnancy as a physiological system through which endogenous anti-fibrotic processes can be leveraged for therapeutic discovery.

**Abstract:**

Cardiac fibrosis is a central determinant of heart failure progression and arises from pathological remodeling characterized by fibroblast activation, myofibroblast differentiation, and excessive extracellular matrix deposition. In contrast, physiological remodeling permits adaptive cardiac growth without net fibrosis. Pregnancy represents an underexplored physiological model of reversible cardiac remodeling. In response to hemodynamic load, the maternal heart undergoes hypertrophic growth that resolves postpartum, constituting a natural paradigm of fibrosis-resistant cardiac adaptation. Pregnancy and lactation are accompanied by profound endocrine and immune reprogramming of maternal tissues. We propose that this hormonal milieu orchestrates coordinated crosstalk among endothelial cells, fibroblasts, and immune cell populations to suppress profibrotic pathways and preserve extracellular matrix homeostasis. Candidate regulators include estrogen, progesterone, prolactin family peptides, relaxin, oxytocin, and components of the renin–angiotensin–aldosterone system. During the postpartum and lactational period, prolactin and oxytocin may further promote reverse remodeling. These hormones likely act by modulating local cytokine and growth factor networks that otherwise drive fibroblast activation. By focusing on non-myocyte cardiac cells and extracellular matrix dynamics, this review positions pregnancy as a translational model to uncover endogenous anti-fibrotic mechanisms and identify novel therapeutic strategies for cardiac fibrosis.

## 1. Introduction

Cardiac remodeling is a dynamic process involving structural and functional changes in the heart in response to physiological or pathological stimuli, which may result in either adaptive or maladaptive responses [1,2,3]. Cardiac remodeling often involves hypertrophy and dilation, and in pathological conditions such as hypoxia, chronic hypertension, or volume overload can lead to fibrosis characterized by excess collagen production and extracellular matrix accumulation [4]. The maternal heart of pregnancy exhibits physiological cardiac enlargement in response to increased hemodynamic load that is reversed during the postpartum period, and normally does not involve a net increase in fibrosis [2,5,6,7]. Understanding the adaptive cardiac changes during pregnancy and postpartum that prevent cardiac fibrosis from occurring may assist in identifying new therapeutic targets to regulate and treat cardiac fibrosis associated with pathological conditions. This narrative review provides an overview of the dual nature of cardiac remodeling, contrasting maladaptive pathological fibrotic remodeling with the non-fibrotic adaptive physiological remodeling of the maternal heart during pregnancy and the postpartum period. We highlight the role of cell–cell communication, especially between fibroblasts and immune cell populations. We examine the hormonal environments that drive adaptive cardiac remodeling during pregnancy and the postpartum period, shaping cellular and extracellular changes of the heart and vasculature. Finally, we discuss our ideas for future directions of peripartum cardiac remodeling research.

## 2. Overview of Cardiac Remodeling

Cardiac remodeling denotes the ensemble of molecular, cellular, interstitial, and geometric alterations that change ventricular size, mass, shape, and pump performance in response to diverse stressors, including hemodynamic changes and tissue injury [1]. Cardiomyocytes make up approximately 75% of the myocardial volume and are organized into a laminar arrangement, aiding in both the efficient mechanical and electrical activity of the heart [4]. The interstitial extracellular matrix (ECM) forms a network between and around the cells. The cardiac ECM mainly comprises collagens, glycoproteins, glycosaminoglycans, and proteoglycans, as well as latent growth factors and proteases [8]. Two broad types of cardiac remodeling are recognized: physiological and pathological. Physiological remodeling occurs in healthy athletes and pregnant individuals, and ultimately, does not cause irreparable alterations to the function or structure of the heart with long-term removal of stressful stimuli [3]. Pathological remodeling can be provoked by ischemic loss of cardiomyocytes, pressure overload driving concentric myocyte growth, and volume overload favoring eccentric chamber dilation, as well as toxic and metabolic stressors [9]. While structural changes in response to these adverse stimuli may initially serve as compensatory adaptations to maintain cardiac output, they often progress to a maladaptive state characterized by dysfunction, heart failure, and increased risk of malignant arrhythmias.

### 2.1. Pathological Cardiac Remodeling

Pathological cardiac remodeling is a complex, multifactorial process involving deteriorating alterations across multiple hierarchical levels of the heart. While initially adaptive, these changes frequently become imbalanced and maladaptive over time, leading to pathological changes in chamber dimensions and cardiomyocyte hypertrophy. Pathological cardiac remodeling is frequently driven by cardiomyocyte apoptosis and other forms of cell death, which secondarily triggers inflammation and fibrosis. These changes lead to scar tissue formation, increased myocardial stiffness, and disruption of electrical conduction [3,10,11]. The structural and functional alterations in pathological remodeling compromise both systolic and diastolic performance, leading to ventricular dysfunction, and progress to heart failure. Heart failure with preserved ejection fraction (HFpEF) and heart failure with reduced ejection fraction (HFrEF) represent phenotypic subdivisions of heart failure based specifically on left ventricular systolic function, as it is the principal determinant of systemic perfusion [11]. Thus, in the context of systemic hemodynamic stress, it is predominantly the left ventricle (LV) that bears the impact and is the primary focus of comparison. However, right ventricular dysfunction may coexist and contribute significantly to disease severity and prognosis [12].

The major compensatory mechanisms for alterations in ejection fraction (EF) are activation of the sympathetic nervous system and renin–angiotensin–aldosterone system (RAAS) as well as release of vasoactive modulators such as endothelin-1 and vasopressin [13]. The effects are an increase in chronotropy, inotropy, systemic vasoconstriction, and renal sodium and fluid retention. The increase in blood volume serves to increase preload and cardiac output, initially resulting in a preservation of EF. However, heightened vasoconstriction increases the afterload and demands the heart to work harder to eject blood, furthering muscle hypertrophy and fatigue, and ultimately leading to reduced EF. Common symptoms of left heart failure include chest pain, cardiomegaly, dyspnea, fatigue, edema, and pulmonary congestion [11].

At the cellular level, cardiomyocytes undergo sarcomeric reorganization. In pressure overload, myocytes add sarcomeres in parallel, resulting in concentric hypertrophy; in volume overload, sarcomeres are added in series, producing eccentric hypertrophy and ventricular dilation [14]. These structural changes are associated with altered metabolic function and, in the long term, reduced contractile efficiency. Concurrently, activation of programmed cell death pathways, including apoptosis and necroptosis, contributes to the progressive loss of viable cardiomyocytes. Because cardiomyocytes are not regenerative, the dead cells are ultimately replaced by ECM [15]. Cardiac fibroblasts play a pivotal role in pathological remodeling as the primary cells responsible for modulation of the ECM. In response to many stressors, fibroblasts differentiate into myofibroblasts, which leads to increased synthesis and cross-linking of type I and III collagen, promoting myocardial fibrosis and increased tissue stiffness [1]. Cardiac fibroblasts also produce matrix metalloproteases that degrade ECM components. The balance between synthesis and degradation of ECM components is critical to ECM homeostasis, which is compromised in pathological cardiac remodeling.

Additionally, in the setting of fibrosis, the coronary microvasculature is compromised through capillary rarefaction, a reduction in microvascular density that impairs oxygen delivery and contributes to ischemia [16]. The hypoxic stress and subsequent elevation in levels of proinflammatory cytokines, such as tumor necrosis factor (TNF) and interleukin 1 beta (IL1B), along with increased production of reactive oxygen species (ROS), perpetuate cellular injury and fibrosis [17]. Collectively, these mechanisms represent an initially compensatory process that becomes increasingly maladaptive, culminating in progressive cardiac dysfunction and heart failure.

### 2.2. Physiological Cardiac Remodeling–Non-Fibrotic Cardiac Adaptation During Pregnancy and Postpartum

Gestational cardiac remodeling in adult females is an excellent model to highlight functional and reversible alterations in the heart that occur without a net increase in fibrosis. During pregnancy, significant hemodynamic changes occur to meet the increased metabolic demands of the developing fetus and placenta [2,5]. Total blood volume expands up to 50%, yielding greater venous return and increased cardiac preload. Cardiac output increases by 30–50%, primarily due to elevated stroke volume and heart rate, peaking in the mid to late second trimester in humans [18]. In response to sustained increased preload, the heart undergoes physiological remodeling with advancing pregnancy, including an increase in mass and chamber size. All four heart chambers enlarge, with the left atrium and ventricle exhibiting the greatest hypertrophy. Gestational steroid hormones, estrogen and progesterone, are largely responsible for the hemodynamic alterations [18]. In addition, the human placenta produces peptide hormones such as chorionic gonadotropin (hCG), relaxin, placental lactogens and oxytocin. Relaxin causes vasodilation [19], while estrogen leads to fluid retention by activation of RAAS mainly by stimulating angiotensinogen production [20]. Increased oxygen demand stimulates erythropoietin release from the kidneys; erythropoietin increases erythrocyte production [19]. This increase in red blood cells is beneficial for both maintaining blood viscosity within the context of increased fluid retention as well as meeting the growing tissue oxygen demand.

**Figure 1:**
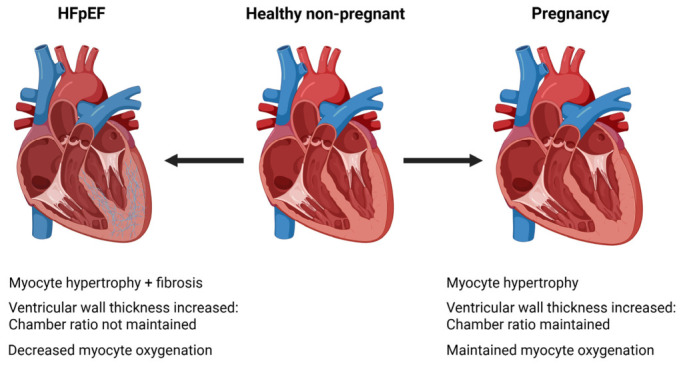
Comparison of cardiac features in normal, HFpEF, and pregnancy states. The normal healthy non-pregnant heart demonstrates typical chamber size, wall thickness, and sufficient oxygenation. HFpEF is characterized by increased left ventricular wall thickness, reduced chamber compliance (stiffness), and fibrosis. In contrast, the heart of pregnancy exhibits physiological remodeling with mild, reversible chamber dilation and proportional wall thickening, allowing increased cardiac output while maintaining normal systolic and diastolic function. EF is preserved in all these states; however, the diastolic dysfunction, resulting from fibrotic deposition in the myocardium, is unique to HFpEF. Because the HFpEF heart cannot fill completely, there is an increased susceptibility for disease progression to include pulmonary hypertension, HFrEF, and MI [12,16,22,24]. Created in BioRender. LaVoie, H. (2026) https://BioRender.com/eg8lm3h (accessed 23 April 2026).

During pregnancy, systemic vascular resistance decreases markedly due to hormonal effects and vasodilation in organs, leading to a reduction in mean arterial pressure, including both systolic and diastolic blood pressure [21]. From a hemodynamic perspective, systemic vasodilation takes place early in pregnancy, lowering afterload, and allowing the heart to function more efficiently despite the heightened volume. The ventricular length to width ratio is mostly preserved despite the hypertrophy [22]. This contrasts with pathological hypertrophy in which the length to width ratio increases. Despite these changes, EF remains mostly preserved throughout pregnancy [7]. These cardiovascular adaptations are considered functional and reversible, enabling the maternal body to effectively support the growing fetus. Importantly, it has been noted that despite the pregnancy-associated cardiac hypertrophy, there is a lack of net fibrosis in contrast to pathological heart failure remodeling [23]. A simplified comparison of HFpEF and pregnancy is shown in Figure 1.

After parturition, most of the cardiovascular alterations of pregnancy slowly revert over the course of weeks to months, although certain structural changes may remain longer, particularly in women who have given birth multiple times [25]. Clapp and Capeless (1997) studied nulliparous and parous women before, during, and after a singleton pregnancy [26]. They found that in both groups, postpartum mean arterial pressure, heart rate, and end-diastolic volume (EDV) gradually approached pre-pregnancy baseline levels but remained significantly different at one year. The study did, however, observe that the magnitudes of these changes were enhanced in parous rather than nulliparous women. Additionally, they observed greater and more rapid increases in ventricular volume and cardiac output, along with a decrease in systemic resistance, in multiparous women compared to nulliparous women up to gestational week 24 [26]. The authors suggested that individuals on their second or third pregnancy were primed physiologically by prior pregnancy experience to adapt more quickly and efficiently to the cardiac demands of gestation. It is important to note that the Clapp and Capeless study recruited mainly athletic women, who are predisposed to physiological hypertrophy.

The structural and functional differences between pregnancy and pathological remodeling are also reflected at the cellular level. As mentioned before, cardiomyocytes undergo hypertrophy during pregnancy; however, this hypertrophy is not linked to fibrosis or cellular disorganization, setting it apart from the pathological remodeling observed in conditions such as hypertension or heart failure. It is hypothesized that controlled alterations in collagen turnover assist in allowing maternal myocardial flexibility as most histological studies measure no net increase peripartum cardiac fibrosis [27,28]. One peripartum study in rats found an altered ratio of type 3 to type 1 collagen protein with the progression of pregnancy which may help explain the reduced stiffness of the maternal heart [29]. Umar and colleagues (2012) compared non-pregnant diestrus, late-pregnant, and 7-day postpartum C57BL/6 female mice. They found that angiogenesis was increased in the heart as well as downregulation of mRNA levels for enzymes that break down the ECM, such as matrix metalloproteinase 2 (MMP2), a disintegrin and metalloproteinase (ADAM) metallopeptidase domain 15 and 17 (ADAM15 and ADAM17). During late pregnancy, mRNA levels for tissue inhibitor of metalloproteinases 1 (TIMP1) have been shown to be consistently increased in the peripartum mouse LV, suggesting its protein might increase to provide temporal inhibition of MMPs [30,31].

A condition where the normal physiological cardiac remodeling fails is peripartum cardiomyopathy (PPCM), characterized by an enlarged, weakened maternal heart with reduced EF that occurs in the last month of pregnancy or the first five months following pregnancy [7]. The etiology of PPCM is unclear but may involve oxidative stress, inflammation, altered hemodynamics, antiangiogenic factors, and abnormal immune responses as well as genetic components. Evidence for inflammation and involvement of immune cells is the finding that patients with PPCM exhibit increased plasma concentrations of interleukin 6 (IL6), TNF, hepatocyte growth factor, C-C chemokine ligand 3 (CCL3), C-X-C chemokine ligand (CXCL) 10, and colony-stimulating factor 1 (CSF1) compared to healthy postpartum and non-postpartum controls [32].

## 3. Etiology of Fibrosis and the Role of Fibroblasts

Fibrosis is a hallmark feature of pathological cardiac remodeling and is driven by the activation of fibroblasts to myofibroblasts, cells that have enhanced contractile activity, similar to smooth muscle cells, and which produce abundant ECM proteins [33]. In the heart, these cells arise predominantly from stress-induced activation of resident, quiescent fibroblasts, although additional sources such as pericytes, endothelial and epithelial transitions have been described [34]. Factors such as inflammation, tissue ischemia, varying mechanical tension, ROS, and age can trigger activation of fibroblasts [35]. The intermediate cell form created during fibroblast transformation to myofibroblasts is known as a proto-myofibroblast. Transforming growth factor beta (TGFB) acts on this transient cell form to facilitate its complete transition to the final myofibroblast phenotype [36], with TGFB1 serving as a major mediator of this transition. The myosin and α-smooth muscle actin (α-SMA) proteins incorporated into the filament proteins of myofibroblasts allow for contractile abilities, important for wound closure [37]. This contracture ability gives the cell a smooth muscle-like phenotype, and it is this characteristic that defines a fully activated myofibroblast [38]. While this response is essential for wound healing and structural repair, persistent activation results in excessive ECM deposition and fibrosis. Herein, we will focus our discussion on the process and mechanisms of cardiovascular fibrosis. Many aspects of fibrosis are common between different tissues and organs; however, there are important details regarding the mechanisms and the functional impact of fibrosis that might be unique in different organs.

### Cardiac Fibrosis

Cardiac fibrosis includes several patterns: reactive interstitial fibrosis, replacement fibrosis, and perivascular collagen deposition [39]. Reactive interstitial fibrosis is caused by persistent hemodynamic stress, which leads to widespread ECM deposition in the absence of considerable myocyte loss, while replacement fibrosis happens following cardiomyocyte death due to ischemia or other insults such as drug toxicity, leading to the development of localized scar tissue [4]. After a myocardial infarction, myofibroblasts produce ECM in not only infarcted tissue but also in healthy tissue distal from the injury, a consequence of the inflammatory response to the acute injury [40]. Additionally, if the stressor persists, myofibroblasts may remain embedded in the scar tissue long after the initial injury response [40]. Despite not having contractile activity, the collagenous tissue is critical for maintaining the chamber’s structural integrity and distributing mechanical stress. Depletion of myofibroblasts in a mouse myocardial ischemia model resulted in reduced collagen production and substantial lethality due to ventricular rupture [41].

Among the ECM proteins produced by myofibroblasts, collagen types I and III are among the most notable. In the early stages of wound healing, the myofibroblasts in the granulation tissue deposit the relatively elastic type III collagen. Type I collagen, the primary type found in end stage fibrosis, is deposited after the granulation tissue is resorbed [42]. It is this type I collagen that is very rigid and makes the cardiac tissue stiff and less compliant. Glycoproteins such as fibrillin, latent TGFB binding proteins, and elastin are also produced; however, it is collagen that alters tissue mechanical properties during fibrosis [42]. This collagen deposition results in decreases in ventricular compliance, EDV, and therefore, stroke volume and cardiac output. Because myofibroblasts deposit scar tissue in place of the damaged cardiomyocytes, the cells are largely pathognomonic for injured cardiac tissue. Cardiac fibrosis contributes to diastolic dysfunction by hindering ventricular relaxation and filling, and to systolic dysfunction by replacing contractile myocardium with non-functional scar tissue. Additionally, fibrosis can lead to disrupted electrical conduction, impaired contractility, and increased risk of arrhythmias [3].

In contrast to these pathological processes, pregnancy-associated cardiac remodeling provides a unique physiological context in which increased hemodynamic load induces ventricular hypertrophy and chamber dilation without increasing net fibrosis, despite the differential expression of ECM proteins in cardiac remodeling specific to gestation. This suggests that, rather than preventing fibroblast activation entirely, pregnancy may involve tight regulation of fibroblast phenotype and ECM turnover, balancing synthesis and degradation to maintain tissue compliance. However, the mechanisms underlying this protection remain incompletely defined. It is unclear whether pregnancy hormones directly suppress myofibroblast differentiation, modulate immune-mediated profibrotic signaling, or alter mechanotransduction pathways within fibroblasts. Importantly, most evidence supporting these protective mechanisms derives from rodent models or in vitro systems, and direct validation in human cardiac tissue is lacking. Furthermore, many studies examine individual pathways, such as TGFB signaling or collagen turnover, in isolation, limiting the ability to construct a unified mechanistic framework. Given that fibrosis arises from the integration of mechanical, inflammatory, and hormonal signals, understanding how pregnancy coordinates these pathways represents a key gap in the field. Elucidating this regulation may provide insight into how pathological remodeling could be redirected toward a more adaptive, fibrosis-resistant state.

## 4. Cell–Cell Communication in Cardiac Remodeling and Immune Cells

As discussed above, fibroblasts respond to a number of different stressors to become activated into myofibroblasts. While the fibroblasts/myofibroblasts are the primary drivers of ECM synthesis and degradation, myocardial remodeling is orchestrated by complex interactions between diverse cells of the heart including resident and transient cells. As mentioned previously, endothelial cells can contribute to fibrosis via conversion to myofibroblasts, a form of endothelial to mesenchymal transition [43]. The hypoxic environment in the setting of myocardial infarction has been shown to promote endothelial to myofibroblast conversion, which contributes to myocardial fibrosis and dysfunction [44]. In addition to direct conversion to myofibroblasts, endothelial cells have been shown to modulate cardiac fibrosis via crosstalk with fibroblasts, largely through secreted factors. For instance, recent studies have illustrated that enhanced expression of the transcription factor Sox9 in endothelial cells promotes heart fibrosis and failure [45]. This is at least partly due to increased expression and secretion of CCN2, a direct gene target of Sox9, which in turn promotes fibroblast migration, activation, and ECM production. On the other hand, endothelial progenitor cells have been shown to release exosomes that can be taken up by fibroblasts and attenuate the fibrotic response [46,47]. In some cases, this anti-fibrotic response is due to particular miRNAs carried in the exosomes derived from endothelial progenitor cells [46]. However, the extent to which endothelial-to-mesenchymal transition contributes quantitatively to the myofibroblast pool in vivo remains controversial, and marker-based approaches may not definitively establish stable lineage conversion.

Communication between cardiomyocytes and fibroblasts can occur via paracrine factors, mechanical junctions, electrical modulators, and ECM interactions [48]. Cardiac fibroblasts have mechanosensitive receptors comprising ion channels and integrins that sense mechanical stress and activate fibrogenic cascades [4]. Herum and colleagues found that stretched cardiac fibroblasts from adult CD1 male and female mice induced a profibrotic phenotype [49]. Additionally, they studied the effects of stretch-induced paracrine signaling in media from cardiomyocytes on cardiac fibroblasts in a co-culture setting. Stretched cardiomyocytes induced proliferation of non-stretched cardiac fibroblasts without cell–cell contact, suggesting paracrine communication between these two cell types through secretions in the shared culture medium. Several signal transduction pathways such as TGFB, endothelin-1, angiotensin II (AngII), connective tissue growth factor, and platelet-derived growth factor have been described to mediate this process [1]. While informative, these in vitro stretch and co-culture systems simplify the in vivo myocardial environment, and may not fully capture the multicellular and biomechanical complexity of remodeling in the injured heart.

Immune mediators can drive temporary scar formation into chronic interstitial and perivascular fibrosis, resulting in ventricular stiffness and eventually heart failure. Immune cells that are known to play a role in cardiac fibrosis include monocytes/macrophages, neutrophils, T and B lymphocytes, mast cells, and dendritic cells [50]. Of all the immune cells, monocytes/macrophages are key orchestrators in both tissue repair and fibrosis [51]. Pinto and team (2016) found through flow cytometry of healthy Cx3cr1GFP/^+^ mouse hearts that cardiac leukocytes comprise 81.4 ± 1.4% myeloid cells (CD11b^+^), 8.9 ± 0.6% B cells (B220^+^), 3.1 ± 0.4% T cells (CD3ε^+^), and 6.6 ± 0.6% non-myeloid/lymphoid (CD11b^−^B220^−^) leukocytes [52]. Alterations in the cardiac leukocyte profile in mouse models of cardiac pathologies include neutrophil invasion contributing to early inflammatory signaling, decline in resident macrophages, inflammatory monocytes and macrophages expansion, and T cell infiltration [53,54,55,56]. We will briefly discuss evidence implicating various immune cell populations in fibrosis and cardiac remodeling.

### 4.1. Monocytes/Macrophages

Monocytes in humans, generally CD14^+^ cells, are a group of antigen-presenting cells which engulf, digest, and process foreign material, and may present the antigens on their cell surface [57]. Monocytes in mice are denoted by the cell marker Ly6C [58]. Monocytes may differentiate into macrophages or dendritic cells, often in the setting of tissue damage and inflammation. The total number of monocytes is generally increased, and many differentiate into macrophages, in the diseased heart. For instance, following myocardial infarction in an experimental mouse model, monocytes rapidly infiltrate the infarcted heart, and the total numbers of monocytes and macrophages are elevated in the first several days after infarction [59]. The mechanisms promoting monocyte recruitment and differentiation in the diseased heart are not completely understood. Resident macrophages, such as cardiac tissue macrophages (cTMs), are essential to the tissue homeostasis. These cTMs are embedded in the interstitial spaces between cardiomyocytes or adjacent to endothelial cells of capillaries [60]. As discussed below, cTMs can be divided into C-C chemokine receptor 2-positive and -negative (CCR2^+^ and CCR2^−^) subsets, that are derived from embryonic and adult hematopoietic lineages, respectively [61]. Studies have illustrated that a subset of cTMs that are CCR2^+^ promote monocyte recruitment to the heart via a myeloid differentiation primary response 88 (MYD88)-dependent mechanism that involves release of monocyte chemoattractant proteins [62]. Other studies have illustrated that the chemokine ligand 1 (CXCL1) promotes monocyte infiltration into the heart in an AngII-induced hypertrophy model [63]. Blocking CXCL1 with a neutralizing antibody attenuated macrophage accumulation in the heart as well as cardiac hypertrophy and fibrosis. Interestingly, CXCL1 and monocytes expressing chemokine ligand 2 (CXCR2), the CXCL1 receptor, were found to be increased in serum from heart failure patients.

Mononuclear phagocytes (monocytes, macrophages and dendritic cells) demonstrate substantial plasticity following tissue injury and can demonstrate opposing effects depending on the type of injury, subpopulation of cells, and time after injury. Macrophages can be classified by a variety of markers or receptors. As mentioned above, CCR2^+^ and CCR2^−^ typically denote proinflammatory and anti-inflammatory subtypes, respectively. CD14 is expressed primarily by monocytes and macrophages while CD16 is expressed by monocytes, macrophages, and some other inflammatory cells [64]. The relative expression of CD14 and CD16 relates to the level of CCR2 expressed on the surface of the cell. High levels of CD14 and no CD16 (CD14^++^CD16^−^) relate to high expression of CCR2. CD14^++^CD16^−^ cells are referred to as classical monocytes. Non-classical monocytes (CD14^+^CD16^++^) have much lower levels of CCR2 [64].

CCR2^+^ monocytes originate from hematopoietic stem cells and are recruited to sites of inflammation, where they differentiate into macrophages [65]. They act as drivers of inflammation, releasing proinflammatory cytokines, further recruiting neutrophils and monocytes, and producing ROS following cardiomyocyte injury. When activated, macrophages produce factors that activate fibroblasts and initiate the process of fibrosis, such as platelet-derived growth factor (PDGF), amphiregulin, TGFB1, IL1B, IL6, IL10, and granulin [66]. Epelman and team (2014) found that in AngII-treated mice that lacked CCR2^+^ macrophages, cardiac IL1B production was abolished [61]. This proinflammatory activity can exacerbate heart failure by promoting further myocardial damage and unfavorable remodeling. Consistent with these mouse studies, elevated numbers of CCR2^+^ macrophages correlate with more severe remodeling in human patients with advanced heart failure [67].

CCR2-negative (CCR2^−^) macrophages originate from embryonic sources such as the yolk sac and fetal liver. They are sustained locally through self-renewal rather than influx to the tissues from circulation [66]. In neonatal development, they support coronary vessel development, cardiac growth, and regeneration. After myocardial injury, CCR2^−^ macrophages help restrain inflammation by inhibiting monocyte recruitment, playing a protective role in cardiac recovery, and preventing myocardial fibrosis after an inflammatory cardiac event [62]. Similar results were found in a transverse aortic constriction (TAC) pressure overload mouse model [68]. Following TAC, macrophages in the heart were increased, in part via expansion of resident CCR2^−^ cells and recruitment of CCR2^+^ cells. Furthermore, preferential ablation of resident cardiac macrophages did not alter TAC-induced cardiac hypertrophy nor EF at post-surgical week one; however myocardial fibrosis was enhanced, and angiogenesis was impaired indicating that resident macrophages normally suppress fibrosis and promote angiogenesis.

Historically, macrophages have been classified into either M1 or M2 activation phenotypes [69]. It has become clear that this is a simplistic view, and macrophages have a range of phenotypes. Broad omics-based approaches have provided substantial insight into these diverse phenotypes and their potential roles in cardiac homeostasis and disease [53]. These approaches are being used to identify macrophage genes that drive cardiac fibrosis. Single-cell RNA-seq (scRNA-seq) was carried out at different times following myocardial infarction in a mouse model [70]. In the early inflammatory phase, these studies confirmed the expansion of Ly6c2^hi^ monocytes and macrophages in the infarcted myocardium. A novel macrophage cluster was identified during the remodeling phase (days 3–7 post-infarction) that was characterized by the expression of arginase 1 (ARG1), fibronectin 1 (FN1) and secreted phosphoprotein 1 (SPP1, osteopontin). Further, these studies illustrated that the chemokine CXCL4 (also known as platelet factor 4) from platelets played an important role in promoting the differentiation of macrophages into the ARG1^+^FN1^+^SPP1^+^, profibrotic phenotype. Studies such as these will advance our understanding of the communication between macrophages and fibroblasts and hopefully help identify novel therapeutic targets to modulate cardiac fibrosis.

### 4.2. Neutrophils

Neutrophils, identified as CD11b^+^CD16^+^CD62L^+^ in humans and CD11b^+^Ly6G^+^ in mice, do not typically have a substantial presence in healthy heart tissue [71]. After an acute myocardial injury, neutrophils arrive within hours as the first immune cells at the damaged site [72]. The cells mainly function to phagocytize debris and release their granules containing enzymes, peptides, and chemokines intended to kill bacteria that may be present, cause tissue destruction, and attract other immune cells [72]. The release of inflammatory mediators, like ROS and proteolytic enzymes, concurrently exacerbates myocardial inflammation and injury [54]. This aggravation of injury often mediates pathological remodeling in the heart. Upon neutrophil death, their cellular remnants are thought to mediate the conversion of macrophages from their proinflammatory phenotype to the anti-inflammatory phenotype [73]. The engulfment of apoptotic neutrophils by macrophages induces the release of the anti-inflammatory IL10 and TGFB as well as pro-resolving lipid mediators [74].

Neutrophilia, or elevated levels of neutrophils in circulation, is associated with poor cardiac outcomes, including higher mortality, in patients in the clinical setting [75]. Antipenko et al. (2024) employed mouse models of myocardial infarction to test whether neutrophil depletion improves cardiac function, employing anti-Ly6G antibody (1A8) and neutrophil-targeted diphtheria toxin strategies [76]. Neutrophil depletion at 4 weeks post-MI significantly improved left ventricular EF, reduced end-systolic volume (ESV) and EDV, decreased cardiac neutrophils and proinflammatory macrophages, and lowered interstitial fibrosis compared to MI controls [76]. These findings demonstrate that targeting neutrophils attenuates adverse cardiac remodeling and fibrosis after myocardial injury. Although these findings suggest therapeutic promise, cell depletion strategies may also disrupt necessary reparative processes, making it difficult to distinguish specific antifibrotic effects from broader alterations in infarct healing.

In humans, blood neutrophils are elevated in PPCM patients (within 3 months postpartum), compared to healthy postpartum and healthy non-postpartum controls [32]. Furthermore, blood lymphocytes in PPCM patients were reduced compared to controls. The healthy postpartum and non-postpartum group cell values were similar. Taken together, a greater neutrophil-to-lymphocyte ratio observed in the PPCM patients indicates increased inflammation with this condition.

### 4.3. T Lymphocytes

T lymphocytes, CD3^+^ leukocytes, are part of the adaptive immune system that helps the body identify and eliminate pathogens as well as infected or abnormal cells. There are two major subsets of T cells, helper (CD4^+^) T cells and cytotoxic (CD8^+^) T cells [77]. While helper T cells have many different subtypes, their collective function is to mediate a specific immune response through cytokine secretion as well as stimulate B cells to produce antibodies. In the inflammatory response, CD4^+^ T cells arrive early in the healing process and help limit infiltration of inflammatory monocytes, regulate inflammation, promote angiogenesis, and support extracellular matrix formation [78].

Mice lacking CD4^+^ T cells show inferior cardiac outcomes and remodeling. Hofmann and team (2012) found that in C57BL/6J wildtype mice, both Foxp3^−^ and regulatory Foxp3^+^ CD4^+^ T cells were activated in a T cell receptor mediated manner in cardiac draining lymph nodes one week after induced-MI. There is also evidence that proliferating T cells are trafficked to injured sites in the myocardium after being activated in the mediastinal lymph nodes [79]. When comparing wildtype mice to CD4 null mice, null mice had a significant increase in leukocytes in the myocardium. The CD4 null mice suffered from LV dilation, decreased quality of scar tissue, and higher mortality. The collagen fibers within the infarcted zone were not only decreased compared to the wildtype mice but also misaligned.

CD4^+^ T cells may also be deleterious in the heart during pathological stress. Laroumanie and team (2014) found that 6 weeks after TAC surgery to induce pressure overload, mice significantly increased the density of both CD4 and CD8 T lymphocytes in cardiac draining lymph nodes [79]. In order to distinguish the effects of each T cell population on cardiac remodeling, they also performed TAC surgeries in MHCII null mice. These mice exhibited ameliorated ventricular dilation, dysfunction, and fibrosis compared to wildtype C57BL/6 and CD8 null mice post-TAC surgery [79]. This suggests that CD4^+^ T cells can have heterogenous effects on cardiac remodeling in different settings of cardiac injury.

CD8^+^ T cells release cytotoxic molecules such as perforin and granzymes, that are necessary to assault microbial membranes and trigger apoptosis [80]. The role of these cells in cardiac remodeling remains mostly unexplored. However, Ilatovskaya and coworkers (2019) studied coronary artery occlusion-induced MI in C57BL/6J and CD8a^tm1mak^ mice, which are deficient in functional CD8^+^ T cells [81]. CD8a^tm1mak^ mice had better survival rates than the control group at seven days post-MI; however, 100% of these mice eventually died of cardiac rupture due to poorly structured scar tissue. There was an obvious acceleration of fibrosis in the CD8a^tm1mak^ mice with collagenous tissue found at both the infarcted location and remote sites. The team cited the upregulated production of soluble collagen as the increase in malformed fibrotic tissue. Additionally, CXCL1, CCL11, MMP2, and matrix metallopeptidase 9 (MMP9) are considered inflammatory markers and were all upregulated in CD8a^tm1mak^ versus the control. Because the mice with nonfunctional CD8^+^ T cells had better survival rates seven days after MI but all eventually died from poorly formed scar tissue, this study indicates the complex stage-dependent roles of this T cell subtype in the setting of cardiac injury.

### 4.4. B Cells

An essential part of the adaptive immune system, B cells, also known as B lymphocytes or CD19/CD20^+^ cells, produce antibodies and cytokines that aid in the body’s defense against infections [82]. These cells have both beneficial and harmful effects following cardiac injury. Goodchild and team (2009) performed an intramyocardial injection of purified bone marrow-derived B cells in the setting of a coronary artery ligation-induced cardiac ischemia [83]. Those B cells preserved cardiac function in male Sprague-Dawley rats, while other bone marrow mononuclear cell types had no protective effect [83]. Ventricular function improved with the injection of B cells, as the fractional ventricular diameter shortening increased from 38% in controls to 44% with fresh B cells and 51% with B cells cocultured with nonhematopoietic stem cells. Additionally, compared to saline control, there were significantly less apoptotic bodies in the heart tissue of rats treated with fresh B cells or co-cultured B cells 48 h after induced MI.

Kallikourdis and coworkers (2017) found that abatacept, a T cell activation blocker, attenuated TAC-induced heart failure in mice by reducing cardiac hypertrophy and the expression of fibrosis-related genes [84]. The protective effect was observed in wildtype mice but not in IL10 knockout mice, unless they were reconstituted with B cells, indicating the necessity of IL10 for protection as well as the competency of B cells to produce the cytokine at sufficient levels to mediate the protective effects and T cell blockage [84].

Autoantibodies against contractile proteins have been associated with inflammatory dilated cardiomyopathy and PPCM. Haghikia and colleagues (2015) measured antibodies against cardiac sarcomeric myosin heavy chain and troponin I (TnI) in the serum of 70 PPCM patients as well as pregnancy-matched healthy women [85]. In PPCM patients, 48% had either one antibody or both in their serum, compared to only 8% in the control group. Additionally, anti-TnI antibodies were associated with pericardial effusion, evidence of increased inflammation, and antibodies against both myosin heavy chain and TnI were associated with reduced LVEF. Overall, patients with autoantibodies had a significantly reduced chance of full recovery of cardiac function postpartum.

### 4.5. Mast Cells

Mast cells (MCs) are innate immune cells of hematopoietic origin and most commonly associated with allergic reactions, notorious for their role in IgE binding and histamine release in Type I hypersensitivity reactions [86]. However, they are abundant in diseased cardiac tissue, including coronary and fibrotic lesions. MCs are often characterized by the composition of its granules, with MCT denoting granules with tryptase only, and MCCT denoting granules with tryptase, chymase, cathepsin G, and carboxypeptidase [72]. There is much debate over whether MCs play a protective or pathogenic role in cardiac remodeling and fibrosis. MCs secrete both pro- and anti-fibrotic cytokines. Tryptase and chymase are activators of TGFB and AngII, both pro-fibrotic mediators [72]. Basic fibroblast growth factor is stored in the granules of mast cells. However, IL10, IL13, CXCL10 and vascular endothelial growth factor A (VEGFA) are all anti-fibrotic and secreted by mast cells. One of the limitations of studying MCs in this setting is that murine animals do not have many mast cells in their cardiac tissue in comparison to humans. Despite these limitations, much evidence exists for a role of MCs in the etiology of cardiac remodeling and fibrosis in humans.

Jaggi and team (2007) studied the effect of a MC stabilizer, ketotifen, on ischemia–reperfusion-induced cardiac injury [87]. Immediately after euthanasia, male Wistar rat hearts were perfused with Krebs–Henseleit solution containing ketotifen and mounted on Langendorff apparatus to be subjected to 30 min of global ischemia and 120 min of reperfusion. Cardiac injury was assessed by lactate dehydrogenase (LDH) and creatine kinase (CK) release as well as infarct size. Ketotifen treated mice showed a significant reduction in the secretion of LDH and CK as well as infarct size in comparison to the control group, indicating reduced cardiac injury with MC stabilization.

Similarly, Levick and team used MC stabilizer nedocromil in spontaneously hypertensive rats [88]. They found that the drug not only decreased macrophage (CD68^+^) infiltration into the myocardium but also reduced LV fibrosis (collagen volume) in comparison to controls. This study provided evidence that MC play a role in hypertension-induced cardiac fibrosis and may influence other immune cells.

Kwon and colleagues (2011) found evidence for a protective effect of MCs in the setting of cardiac stress [89]. Cardiomyocytes treated with MCs survived longer in hypoxia than the untreated control group. These findings were significant, as was the decrease in Troponin I secretion in MC-treated cardiomyocytes versus the control in the same hypoxic setting. MC-treated rat cardiac microvessel endothelial cells in vitro displayed significant increases in tubal density as well as tubal elongation lengths, measurements for angiogenesis, compared to control cells.

These studies indicate why it is difficult to conclusively categorize MCs as protective or deleterious in cardiac remodeling due to their complex, context-dependent roles in the heart. MCs can contribute to tissue repair, angiogenesis, and modulation of inflammation, supporting protective remodeling after myocardial injury. Yet, they are also implicated in promoting fibrosis, adverse ventricular remodeling, and chronic inflammation, which can worsen cardiac function. Experimental models have yielded conflicting results and therefore, more studies need to be done in models more representative of cardiac MC density and function in humans. Interpretation of mast cell studies is further complicated by major species differences in cardiac mast cell abundance and distribution, which may limit direct extrapolation from rodent models to human disease.

### 4.6. Limitations to Cell–Cell Communication Studies

Cell–cell communication studies support a multifactorial model of cardiac fibrosis, but mechanistic evidence varies by cell type and model. Much of the evidence for cardiomyocyte-fibroblast crosstalk derives from in vitro stretch and co-culture models that cannot fully reproduce the spatial, stiffness, neurohormonal activation, and inflammatory microenvironment of the myocardium. Interpretation of studies is complicated by the temporal and functional plasticity of immune responses. Immune populations often have stage-dependent roles, contributing to early repair but promoting fibrosis when activation is prolonged or excessive. Translational relevance is limited by the heavy reliance on rodent models of myocardial infarction, TAC, and AngII, inconsistent consideration of biological sex, and predominantly associative human data. Future work should therefore prioritize spatial and single-cell profiling, lineage-tracing approaches, and validation in human tissue. Human induced pluripotent stem cells (iPSCs) have been used to generate cardiac organoids [90]; such iPSC models may serve as a future approach to gain knowledge of human cardiac cell interactions and study variations in the cytokine and hormone milieu.

## 5. Hormones in Cardiac Remodeling During Pregnancy and the Peripartum Period

As discussed above, the cardiac remodeling of normal pregnancy and the postpartum period does not lead to a net increase in fibrosis in the maternal heart. Since hormones are the dominant regulators of the physiological changes during pregnancy, they likely contribute to the beneficial cardiac remodeling and the lack of cardiac fibrosis. The major hormones during pregnancy or in the peripartum period are estrogen(s), progesterone, relaxin, prolactin and prolactin-like proteins, hCG (in humans), and oxytocin [91].

A major limitation in understanding pregnancy-associated cardiac remodeling is the inability to directly investigate the cardiac-hormone-immune-fibroblast axis in humans. Ethical constraints preclude invasive sampling of cardiac tissue during pregnancy, restricting most human studies to indirect assessments such as imaging, circulating biomarkers, or postpartum analyses [92]. As a result, the dynamic interplay between hormonal signaling, immune cell phenotype, and fibroblast activation within the ventricular myocardium remains largely inferred rather than directly demonstrated. Furthermore, human pregnancy is inherently heterogeneous, influenced by genetic variability, parity, metabolic status, and comorbidities, which complicates the interpretation of immune and hormonal contributions to remodeling and limits the ability to establish causality [7,93]. Importantly, many proposed mechanisms, particularly those suggesting that pregnancy hormones actively suppress fibrosis through modulation of immune-fibroblast interactions, remain largely correlative and lack direct experimental validation in human systems.

Rodent models have, therefore, been indispensable for mechanistic investigation, as they enable controlled manipulation of hormonal pathways, immune cell populations, and fibroblast signaling in vivo [7]. Genetic tools such as lineage tracing, conditional knockout models, and inducible receptor systems have provided key insights into how hormonal cues may regulate immune cell recruitment and fibroblast activation during cardiac remodeling [41,94]. However, these models introduce significant limitations when applied to the study of pregnancy.

**Figure 2:**
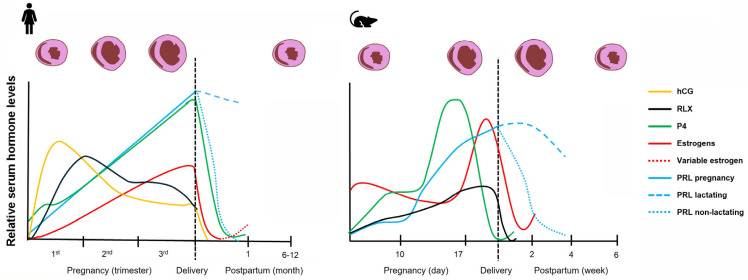
Profiles of pregnancy hormones during gestation that may contribute to cardiac remodeling. (**Left**) In humans, progressive increases in estrogen (E2 and E3) and progesterone drive physiological ventricular remodeling characterized by eccentric hypertrophy, chamber dilation, and increased cardiac output (depicted above graph). Prolactin rises during gestation and remains elevated postpartum during lactation, while relaxin supports adaptive remodeling by enhancing vascular compliance and regulating extracellular matrix turnover. Cardiac structural changes that occur during pregnancy are mostly reversed within 6–12 months postpartum. During the postpartum period estrogen levels vary with lactation. Postpartum E3 is negligible and E2 remains low during intense periods of lactation and returns to cyclic levels as lactation decreases. (**Right**) In rodents (mice and rats), estrogen (E2) and progesterone increase throughout pregnancy, but serum progesterone falls off before parturition due to its increased metabolism. Prolactin rises throughout pregnancy, and its persistence postpartum depends on lactation status. Relaxin peaks in mid-to-late gestation but returns to baseline after birth. For clarity, placental lactogens and oxytocin are omitted from the graphs. Placental lactogen hormone types vary by species, increase during pregnancy, and disappear with placental loss. Oxytocin surges around parturition, and postpartum episodic increases in oxytocin in both human and rodents are associated with offspring nursing activity. Values are shown as relative trends rather than absolute concentrations. Abbreviations: hCG, human chorionic gonadotropin; RLX, relaxin; P4, progesterone; PRL, prolactin; E2, estradiol; E3, estriol. Graphs adapted from data in references [2,6,7,18,19,95,99,100,101,102,103,104].

Species-specific differences in endocrine regulation result in distinct hormonal environments that may differentially influence immune and fibroblast behavior. In humans, the ovarian corpus luteum is the major source of estrogen and progesterone until the eighth week of gestation, at which time the placental trophoblasts assume this function for the remainder of pregnancy. In contrast, rodent pregnancy depends on ovarian luteal progesterone production throughout gestation, with a shift from ovarian to placental estrogen synthesis. Rodent placentas express 17-hydroxylase which allows synthesis of androgens from progestins, which are subsequently used to produce estrogens via placental aromatase [95]. This differs from the human placenta which produces estrogen, mainly estriol, by cooperation of the fetal adrenal and liver in providing androgen substrate to placental aromatase. In women, estrogen, progesterone and prolactin continually rise until parturition, after which the levels of all pregnancy hormones decline rapidly to pre-pregnancy levels, except for that of prolactin which remains elevated in lactating women. In comparison, rodent serum progesterone levels drop near the end of pregnancy, which facilitates parturition. Figure 2 provides a graphical summary of pregnancy and postpartum hormonal changes in humans and mice along with accompanying changes in heart size.

Additionally, differences in immune system composition, placental structure, gestational timing, and hemodynamic load complicate direct translation of findings. Pregnancy averages between 21 and 23 days in laboratory rats and 19–21 days in mice, with average litter sizes between 6 and 12 and 6 to 8 pups, respectively [96,97]. These larger litter sizes may exaggerate physiological stress amplifying rodent remodeling responses that are not fully reflective of human physiology. Lactation in laboratory rodents typically lasts 21 days. In contrast, human gestation length is approximately 266 to 280 days with singleton pregnancies being the most common, and lactation duration in women can be highly variable or even prevented [98]. Data supporting a cardioprotective role for pregnancy hormones in the peripartum period are limited due to experimental limitations. Most experimental data is derived from rodent pregnancy models or in vitro studies of isolated cardiac or vascular cells. In addition, conclusions regarding hormone effects have often been inferred from studies of male and non-pregnant female rodents.

Critically, most current models examine hormonal, immune, and fibroblast pathways in isolation, whereas pregnancy represents a highly integrated system in which these components interact dynamically over time. This fragmentation limits the ability to define a cohesive mechanistic framework for how pregnancy hormones coordinate immune cell function and fibroblast activity to prevent fibrosis. Moving forward, there is a clear need for integrative approaches that combine rodent mechanistic studies with emerging human data. Such efforts will be essential to determine whether the protective features of pregnancy-associated remodeling can be leveraged for therapeutic strategies targeting fibrosis and heart failure.

### 5.1. Estrogen

The steroid hormone estrogen has been shown to have beneficial effects on the heart and vasculature in model systems. Estrogens in humans and rodents include three major estrogen molecules: estrone (E1), 17-beta estradiol (E2), and estriol (E3). E2 is the dominant form in cycling humans and rodents and the major estrogen during rodent pregnancy, whereas E3 is the dominant estrogen of human pregnancy once placental steroidogenesis is established. Estrogens signal through two nuclear receptors estrogen receptor alpha (ESR1, ERα) and estrogen receptor beta (ESR2, ERβ) and a G protein-coupled estrogen receptor associated with the membrane, GPER [105].

In non-pregnant mice, estrogen exerts cardioprotective effects in part by altering myocardial β-adrenergic signaling and calcium levels. Normal activation of cardiomyocyte catecholamine β1 adrenergic receptor (ADRB1) acutely increases heart rate and contractility [106]. Chronic ADRB1 activation or its overexpression exerts maladaptive effects on cardiac structure and function through prolonged cellular cAMP signaling and calcium overload. Chu et al. (2006) showed that the hearts of ovariectomized female rats had increased ADRB1 and CACNA1C (voltage-gated L-type Ca^2+^ channel) and decreased levels of the sodium-calcium exchanger NCX [107]. Furthermore, estradiol benzoate treatment normalized these protein levels. A second study found that ovariectomized mice downregulated sarcoplasmic reticulum Ca^2+^-ATPase (SERCA) level and activity; SERCA lowers cytoplasmic Ca^2+^ after cardiac muscle contraction [108]. Additionally, estrogen acts in part by downregulating ADRB1, providing a more balanced ADRB1 to ADRB2 ratio favoring ADRB2 coupling to G_i_ which is antiapoptotic [109].

Estrogen is protective in mouse models of cardiac hypertrophy, such as that induced by TAC or AngII. Wildtype male mice with TAC demonstrate more cardiac hypertrophy and greater signs of heart failure compared to non-pregnant wildtype female mice. With TAC, the male mice exhibited a higher induction of matrix-remodeling genes, repression of mitochondrial genes in the cardiac tissue, and greater cardiac fibrosis [110]. In comparison, male and female differences in response to TAC were negligible in ESR2 null mice implicating ESR2 in the protective action in females. Furthermore, using AngII to induce cardiac hypertrophy and fibrosis in ovariectomized female mice, E2 administration to wildtype mice attenuated cardiac fibrosis whereas ESR2 null mice were not protected [111]. Taken together, these studies illustrate that the cardioprotective benefits of E2 against pathological hypertrophy and fibrosis occur through ESR2.

There is also strong evidence in vascular models that estrogen has vasoprotective properties. This is due in part to its ability to increase the expression and activation of endothelial nitric oxide synthase (eNOS), which produces nitric oxide, a potent vasodilator [112]. For example, ovariectomized mice without E2 treatment displayed decreased pial artery expression of eNOS with an increase in caveolin-1 (CAV1), an eNOS inhibitor and calcium channel regulator. In contrast, in the same study, ovariectomized mice treated with E2 saw a complete restoration of eNOS levels and a decrease in CAV1 [112]. Vascular smooth muscle cell (VSMC) over-proliferation can cause medial thickening; estrogen has a benefit by inhibiting VMSCs proliferation [113]. It is conceivable that the vasoprotective effects of estrogen may contribute to its beneficial effects on cardiac remodeling and fibrosis during pregnancy and postpartum.

### 5.2. Progesterone

In addition to estrogen, progesterone (P4), another steroid hormone, is also critical for maintenance and progression of pregnancy through its actions on the endometrium, vasculature, and immune system [5]. Progesterone receptors (PGRs) include nuclear receptors (nPGR-A and nPGR-B), membrane progesterone receptors (mPGR), and mitochondrial PGR (PGR-M) [114]. P4 influences the cardiovascular system via multiple mechanisms [115]. Similar to estrogen, progesterone exerts a vasodilatory effect through the activation of eNOS-mediated nitric oxide production [116]. Moreover, progesterone shows anti-inflammatory and anti-fibrotic activity which involves inhibition of nuclear factor kappa B (NFKB) and TGFB [117]. Dubey et al. (1998) showed that estrogen, and its derivatives, as well as progesterone independently inhibited the proliferation of cardiac fibroblasts in vitro [118]. In pregnancy, progesterone opposes RAAS-driven aldosterone effects at its receptor, enabling volume expansion without the hypertensive consequences [119].

Dai and colleagues (2019) found evidence of mitochondrial PGR-M modulation of cardiac beta-oxidation and remodeling in mice [120]. PGR-M is not natively found in rodents, only in humans and nonhuman primates. Using a tetracycline inducible system to express cardiac PGR-M in mice, females that underwent TAC surgery displayed a ligand-dependent decrease in heart failure following 4 weeks of progesterone treatment. This was evident by the downregulation of brain natriuretic peptide, a protein that is released in response to cardiomyocyte stretch, as well as the upregulation of sarcolipin, which disrupts SERCA and calcium entry into the sarcoplasm reticulum [120]. Furthermore, genes involved in nutrient metabolism were significantly enriched in mice expressing PGR-M, alongside upregulated myogenic pathways, including sarcomere development, ATPase activity, and energy production.

Further evidence for progesterone action in the heart was provided by Goldstein and team (2004) using young adult ovariectomized Sprague-Dawley rats [121]. Progesterone supplementation to ovariectomized rats exhibited a significant increase in cardiac protein synthesis compared to sham mice, ovariectomized mice with E2 alone or E2 and P4. In addition, P4 supplementation to ovariectomized animals led to a 20% increase in plasma volume compared to unsupplemented controls [121]. Furthermore, P4 receptor antagonist, RU486, attenuated the increase in protein synthesis; however, RU486 did not return plasma volume to the control levels indicating that the increase in plasma volume was not the initiator for increased cardiac protein synthesis and suggests an alternative site for progesterone action.

### 5.3. Relaxin

Relaxin is a peptide hormone involved in multiple maternal physiological adaptations during pregnancy, with its most recognized action being modulation of the ECM in the female reproductive tract to promote tissue softening and parturition [122]. Relaxin is secreted by the corpus luteum, uterus, and placenta. Humans have relaxin 1, 2, and 3 while rodents only have relaxin 1 and 3 [101]. In humans, relaxin levels rapidly rise and peak by the end of the first trimester and decrease afterwards to an intermediate level, while rats have a distinct rise in concentrations after gestation day 8 or 9 and rise continuously to the end of pregnancy (Figure 2) [101].

Relaxin receptors (RXFP family) are expressed in many tissues such as brain, heart, blood vessels, kidneys, muscle, and bone [122]. Relaxin contributes to the hemodynamic changes of pregnancy through its effects as a systemic and renal vasodilator. In non-pregnant female rats, human recombinant relaxin delivery increased cardiac output, renal blood flow, and glomerular filtration rate [123].

The functions of relaxin are not limited to pregnancy, and studies in non-pregnant animals indicate relaxin mediates ECM remodeling, vasodilation, and angiogenesis, and has anti-fibrotic and anti-inflammatory effects [101,124]. Conrad and team (2011) found that relaxin reduced systemic vascular resistance and pulsatile arterial load by reducing vessel wall stiffness [101]. This effect is mediated through increased nitric oxide synthesis and VEGF activity. Samuel and coworkers (2004) noted the protective effects of relaxin against fibrosis [124]. Relaxin was shown to inhibit AngII and insulin-like growth factor 1-mediated fibroblast proliferation by up to 50% in 1-day-old Sprague Dawley rats. Additionally, relaxin decreased expression of α-SMA by up to 88% and collagen by up to 80% in cardiac fibroblasts treated with AngII and TGF-β, indicating relaxin’s attenuation of these initiators of fibroblast differentiation [124].

### 5.4. Prolactin and Placental Lactogens

Prolactin and placental lactogens are structurally similar polypeptide hormones, and target cytokine-like membrane receptors which act through JAK-STAT signaling pathways [125]. Two forms of the prolactin receptors exist, a long form which mediates most of prolactin function and an alternatively spliced short form whose function is less clear. Prolactin is synthesized in the anterior pituitary gland, central nervous system, immune system, uterus, placenta, and the mammary glands. Pituitary prolactin is tonically inhibited by hypothalamic dopamine, and estrogen influences prolactin levels [126]. Males and non-pregnant females typically have low basal levels of prolactin, as its main function is to facilitate mammary gland development and milk production. In postpartum females, the trigger for prolactin synthesis for milk production is activation of sensory nerves in the nipple by mechanical stimulation such as suckling [125]. When a female is no longer lactating, prolactin levels return to pre-pregnancy basal levels.

In addition to its prevalent effects on lactation, prolactin can influence immune cell function [127]. The prolactin receptor is expressed in lymphocytes, macrophages, natural killer cells, and granulocytes [128]. Prolactin has been found to not only have receptors on T and B lymphocytes but also to be secreted by these immune cells [129]. Prolactin’s relationship to immune cell phenotype and function in the maternal heart during pregnancy is largely unexplored. Angiogenic imbalance, particularly towards anti-angiogenesis, is thought to be a major contribution to PPCM [130]. Cleaved prolactin has been proposed to have a role in PPCM based on studies in rodents, where experimentally elevated levels of a truncated anti-angiogenic and proapoptotic form of prolactin (16 kDa prolactin) are present mice with a PPCM phenotype [131]. Dopamine agonists such as bromocriptine, which reduce prolactin secretion and all its circulating forms have been shown to be protective against PPCM [132]. Whether the protective effect of blocking prolactin secretion is due to reducing the volume load on the heart by ceasing lactation, altering immune cell function or a direct effect of reducing prolactin anti-angiogenic cleavage product is unclear. Yet, among the diverse potential etiologies, it is critical to note that PPCM exhibits the most favorable prognosis clinically for myocardial recovery among the diverse etiologies of heart failure [133]. Therefore, despite these limitations of the studies at present, this fact of improved recovery compared to other cardiomyopathies underscores the importance of elucidating PPCM’s underlying mechanisms, as a clearer understanding of its pathophysiology is essential for the development of targeted and effective therapeutic strategies.

Human placental lactogen (hPL) is a hormone secreted by the syncytiotrophoblasts in the placenta during pregnancy [134]. hPL is the most abundantly produced placental hormone and plays a vital role in pregnancy and fetal support. It aids in the mammary gland alveolar and duct development and is important for maternal metabolism of carbohydrates and lipids, supplying the nutrients for fetal development [134,135]. However, there is little evidence of a role for hPL in cardiac remodeling or cardiomyopathy recovery.

### 5.5. Human Chorionic Gonadotropin

hCG is the hormone well-known for pregnancy detection in humans and corpus luteum survival. It is a heterodimeric glycoprotein that binds the luteinizing hormone-chorionic gonadotropin receptor (LHCGR) which is a seven-transmembrane domain G-protein couple receptor which can also bind luteinizing hormone (LH) [136]. In humans, hCG is produced by trophoblast cells, and its serum levels peak in the first trimester. Its main role is stimulating receptors on the corpus luteum to produce P4 until the placenta takes over this function [137]. hCG contributes to trophoblast invasion of the endometrium and aids in placental–fetal nutrient transportation by stimulating angiogenesis and vascular remodeling. There is also some evidence in humans that hCG has direct effects on the immune system [138]. hCG targets uterine natural killer cells, regulatory T cells, and B cells, helping prevent rejection of the conceptus; furthermore, hCG has also been suggested as an instigator to gestational insulin resistance [135]. There is limited evidence supporting a role for hCG in cardiac remodeling during pregnancy in humans. In contrast, rodents do not make hCG, and prolactin is the luteotrophic factor in those species with the support of LH. LH in rodents could potentially have effects on the immune system similarly to hCG, as both these hormones act through the same receptor; however, most evidence suggests LH/hCG effects are indirect through their stimulation of steroid hormone levels [139].

### 5.6. Oxytocin

Oxytocin is a nonapeptide hormone made mainly by the hypothalamus and released from the posterior pituitary but is also made and released to lesser degrees by the placenta, uterus and mammary glands [103]. Oxytocin’s major targets are in the reproductive system, oxytocin receptors (OXTRs), which are also found in the heart and vasculature [140]. Levels of oxytocin slowly rise during pregnancy until there is a surge around the time of parturition that causes uterine contractions. Postpartum oxytocin helps to expel the placenta, further contract the uterus, and reduce postpartum bleeding by constricting uterine blood vessels. Postpartum oxytocin levels vary with lactation, and nursing events induce oxytocin pulses. During pregnancy, estrogen increases OXTRs in target tissues to aid in vascular system accommodation and preparation of the myometrium for parturition.

Oxytocin has been shown to exert cardioprotective effects even in non-pregnant animals. Oxytocin reduces blood pressure and heart rate in part by increasing atrial natriuretic peptide release and decreasing sympathetic tone [140]. Furthermore, it promotes glucose uptake by cardiomyocytes, which may provide these cells with energy to resist stress. Oxytocin treatment inhibited Ang II-induced hypertrophic changes in cardiomyocytes in vitro and in mice in vivo, supporting a protective role against pathological cardiac growth [141]. Oxytocin protected against ischemia and reperfusion injury in a rat model, an effect associated with reduced mast cell degranulation [142]. In addition, oxytocin influenced the release of proinflammatory cytokines like TNF and IL6 and enhanced anti-inflammatory cytokines like IL10 during cardiac injury [140]. In summary, oxytocin effects are wide reaching, and additional studies are needed to clarify its cardioprotective role during the peripartum period.

## 6. Future Directions

Although cardiac remodeling during pathological and physiological conditions has been studied to varying degrees, large knowledge gaps remain particularly regarding pregnancy-induced cardiac remodeling. What drives the cardiac remodeling occurring during pregnancy that allows it to reverse postpartum? During pregnancy, the hypertrophy of cardiomyocytes is accompanied by phenotypic changes in other cardiac cells including fibroblasts, endothelial, and immune system cells [143]. How the key hormonal influences of E2, P4, prolactin family molecules, relaxin, and RAAS integrate at the cellular level to drive cardiac cellular phenotype change during pregnancy is poorly understood. Just as important as understanding the onset and maintenance of cardiac changes during pregnancy is elucidating the regulation of reverse cardiac remodeling in the postpartum period and the impact of lactation. In the postpartum period, it is unknown if myocytes, fibroblasts, and ECM are actively changing or whether remodeling is due merely to the removal of pregnancy hormones and restoration of normal hemodynamic load. There is limited data in rodents that indicate collagen type III levels increase while collagen type I levels decrease during pregnancy and remain so at one week postpartum, suggesting active ECM remodeling during both periods [29]. However, most studies using picrosirius red staining of pregnant and postpartum hearts to detect type I and type III collagen show no increase in total collagen levels supporting the hypothesis that synthesis and turnover are coordinately regulated to prevent excess ECM accumulation in the pregnant heart [27,28]. Given the complexity of collagen synthesis and assembly, analyses of collagen isoforms, orientation and cross-linking, and their regulatory molecules are needed for cardiac tissue during pregnancy and postpartum to better understand matrix remodeling.

**Figure 3:**
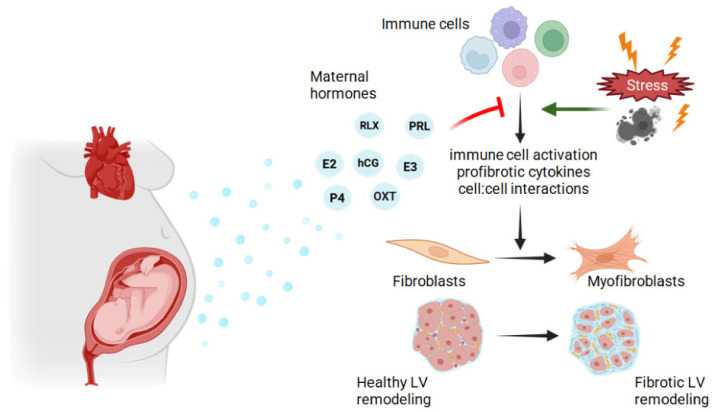
Simplified model of maternal hormone control of cardiac remodeling during pregnancy and the peripartum period. It is hypothesized that maternal pregnancy and peripartum hormones modulate immune cell and fibroblast responses to increased cardiac load during pregnancy leading to beneficial cardiac adaptation. In pathological conditions, cellular stress leads to inflammation, immune cell activation, secretion of proinflammatory cytokines, and cell–cell interactions that promote fibrosis through excess extracellular matrix deposition by myofibroblasts. Pregnancy hormones cooperate to control immune cell responses to maternal cardiac volume overload, stress, blocking these adverse responses and thereby preventing fibroblast transition into myofibroblasts. These coordinated interactions support adaptive cardiac remodeling with minimal fibrosis. Abbreviations same as in Figure 2. OXT, oxytocin. Image created in BioRender. LaVoie, H. (2026) https://biorender.com/cd5phxr (accessed 23 April 2026).

Pregnancy is a special immunological state designed to tolerate the development of a foreign fetus while still protecting the mother from foreign pathogens. Could immunological changes of pregnancy extend beyond the maternal–fetal interface to the heart, suppressing activation of immune cells that could contribute to fibroblast activation? While Lintao et al. (2023) described the presence of fetal immune cell populations in the maternal mouse heart during pregnancy and postpartum, little is known regarding the immune cell populations in the heart throughout pregnancy and postpartum [144]. Further studies defining the immune cell population, evaluating immune cell interactions with other cardiac cells, including via cell–cell contact and cellular secretions (i.e., cytokines, growth factors) in the female heart during pregnancy and postpartum are desperately needed. Figure 3 summarizes our hypothetical model of pregnancy hormone, fibroblast and immune cells interactions during pregnancy and the peripartum period that prevent fibrosis.

## 7. Conclusions

In conclusion, distinguishing between physiological and pathological cardiac remodeling is essential for understanding the divergent mechanisms that drive adaptive versus maladaptive cardiac remodeling. Physiological cardiac remodeling associated with natural stressors such as exercise or pregnancy, involves reversible changes and balanced neurohormonal responses. In contrast, pathological cardiac remodeling and heart failure are marked by persistent stress, chronic inflammation, and sustained action of RAAS, all of which contribute to irreversible fibrosis and disease progression. When examining the broad spectrum of diseases associated with cardiac fibrosis, there is some overlap of shared pathophysiological molecular signatures. For instance, common to heart failure and PPCM are elevated levels of plasma biomarkers IL6, TNF, asymmetric dimethylarginine, and cathepsin D [145,146,147,148,149,150]. sFLT1 is another factor often increased in PPCM and other forms of heart failure, but its source varies. In nongestational-related cardiomyopathy, the vascular endothelial cells and monocytes produce sFLT1, yet placental trophoblasts produce the majority of sFLT1 in PPCM [130,151,152]. However, a commonality of biomarkers does not necessarily imply identical pathogenesis. Instead, they may reflect convergent downstream signaling, such as impaired angiogenesis, oxidative stress, and fibroblast activation, which ultimately drive cardiac fibrosis and dysfunction. From a translational perspective, this raises both opportunities and challenges. Targeting shared pathological cascades could have broad therapeutic relevance across heart failure and PPCM. Yet, the organ-specific origin of these factors suggests that effective interventions may need a more refined strategy. Despite advances in medical treatment, heart failure remains a leading cause of morbidity and mortality worldwide, while evidence shows fibrosis is irreversible and contributes significantly to cardiac dysfunction. Current treatment options often provide only symptomatic relief or modest improvements in disease progression, leaving many patients with dire long-term prognosis. Pregnancy offers a model to study reversible cardiac changes and broaden our understanding of the complex interplay between different organ/cell systems in cardiac remodeling. Identification of hormone regulated biomarkers of fibrosis prevention from pregnancy and postpartum models could lead to identification of novel druggable targets. A deeper understanding of these complex interactions holds promise for the development of targeted therapies that can mitigate pathological remodeling while preserving or enhancing beneficial adaptive responses.

## Data Availability

No new data were created or analyzed in this study.

## Abbreviations

The following abbreviations are used in this manuscript:

ADAM	a disintegrin and metalloproteinase
ADAM15	ADAM metalloproteinase domain 15
ADAM17	ADAM metalloproteinase domain 17
ADRB1	beta-1 adrenergic receptor
ADRB2	beta-2 adrenergic receptor
AngII	angiotensin II
ARG1	arginase 1
CAV1	caveolin-1
CD	cluster of differentiation
CK	creatine kinase
CCL3	C-C chemokine ligand 3
CCL11	C-C chemokine ligand 11
CSF1	colony stimulating factor 1
cTMs	cardiac tissue macrophages
CXCL1	chemokine ligand 1
CXCL2	chemokine ligand 2
CXCL4	platelet factor 4
CXCL10	chemokine ligand 10
E1	estrone
E2	17-beta estradiol
E3	estriol
ECM	extracellular matrix
EDV	end-diastolic volume
EF	ejection fraction
eNOS	endothelial nitric oxide synthase
ERα	estrogen receptor alpha
ERβ	estrogen receptor beta
ESR1	estrogen receptor 1
ESR2	estrogen receptor 2
ESV	end-systolic volume
FN1	fibronectin 1
GPER1	G protein-coupled estrogen receptor 1
hCG	human chorionic gonadotropin
HFpEF	heart failure with preserved ejection fraction
HFrEF	heart failure with reduced ejection fraction
hPL	human placental lactogen
IL1B	interleukin 1 beta
IL6	interleukin 6
IL10	interleukin 10
IL13	interleukin 13
IPSCs	induced pluripotent stem cells
LDH	lactate dehydrogenase
LH	luteinizing hormone
LV	left ventricle
MCs	mast cells
MCT	mast cell subtype with tryptase-only granules
MHCII	major histocompatibility complex class II
miRNAs	microRNAs
MMP	matrix metalloproteinase
MMP2	matrix metalloproteinase 2
MMP9	matrix metalloproteinase 9
mPR	membrane progesterone receptor
mRNA	messenger RNA
MYD88	myeloid differentiation primary response 88
NCX	sodium-calcium exchanger
NFKB	nuclear factor kappa B
nPGR-A	nuclear progesterone receptor A
nPGR-B	nuclear progesterone receptor B
OXTRs	oxytocin receptors
P4	progesterone
PDGF	platelet-derived growth factor
PPCM	peripartum cardiomyopathy
PGR-M	mitochondrial progesterone receptor
PGRs	progesterone receptors
RAAS	renin–angiotensin–aldosterone system
ROS	reactive oxygen species
RU486	mifepristone
RXFP	relaxin family peptide receptor
scRNA-seq	single-cell RNA sequencing
SERCA	sarcoplasmic reticulum calcium ATPase
SPP1	secreted phosphoprotein 1 (osteopontin)
TAC	transverse aortic constriction
TGFB	transforming growth factor beta
TGFB1	transforming growth factor beta 1
TIMP1	tissue inhibitor of metalloproteinases 1
TNF	tumor necrosis factor
TnI	troponin I
Treg	regulatory T cells
VEGF	vascular endothelial growth factor
VEGFA	vascular endothelial growth factor A
VMSC(s)	vascular smooth muscle cell(s)
α-SMA	alpha-smooth muscle actin
